# Supplementary material for: Fishing for newly synthesized proteins with phosphonate-handles
Source: Nat Commun. 2020 Jun 26;11:3244. doi: 10.1038/s41467-020-17010-0 (PMC7320153; doi:10.1038/s41467-020-17010-0)
Supplement: Supplementary file 1 — Supplementary Information [file 41467_2020_17010_MOESM1_ESM.pdf]

Fishing for newly synthesized proteins with phosphonate-handles

Kleinpenning et al.

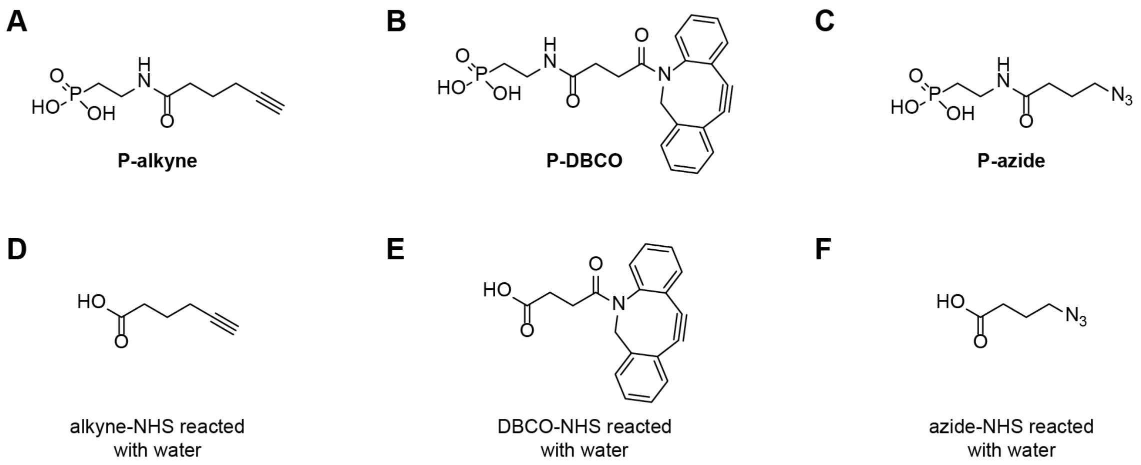

Supplementary Figure 1: Structures of the three P-labeled probes and potential hydrolysis byproducts. (A) P-alkyne: acetylene-containing phosphonate for CuAAC reactions. **(B) P-DBCO**: phosphonate functionalized with dibenzocyclooctyne for SPAAC reactions. **(C) P-azide**: phosphonate modified with an azide for CuAAC reactions. **(D-F)** Side products that may be produced during the synthesis of the P-labeled probes due to hydrolysis of the starting material. Less than 2.5% of the peptides were found to be reacted with the byproducts, 100% is the sum of unmodified, azide- or alkyne-labeled peptides, phosphonate-labeled peptides and byproducts (Supplementary Figure 2).

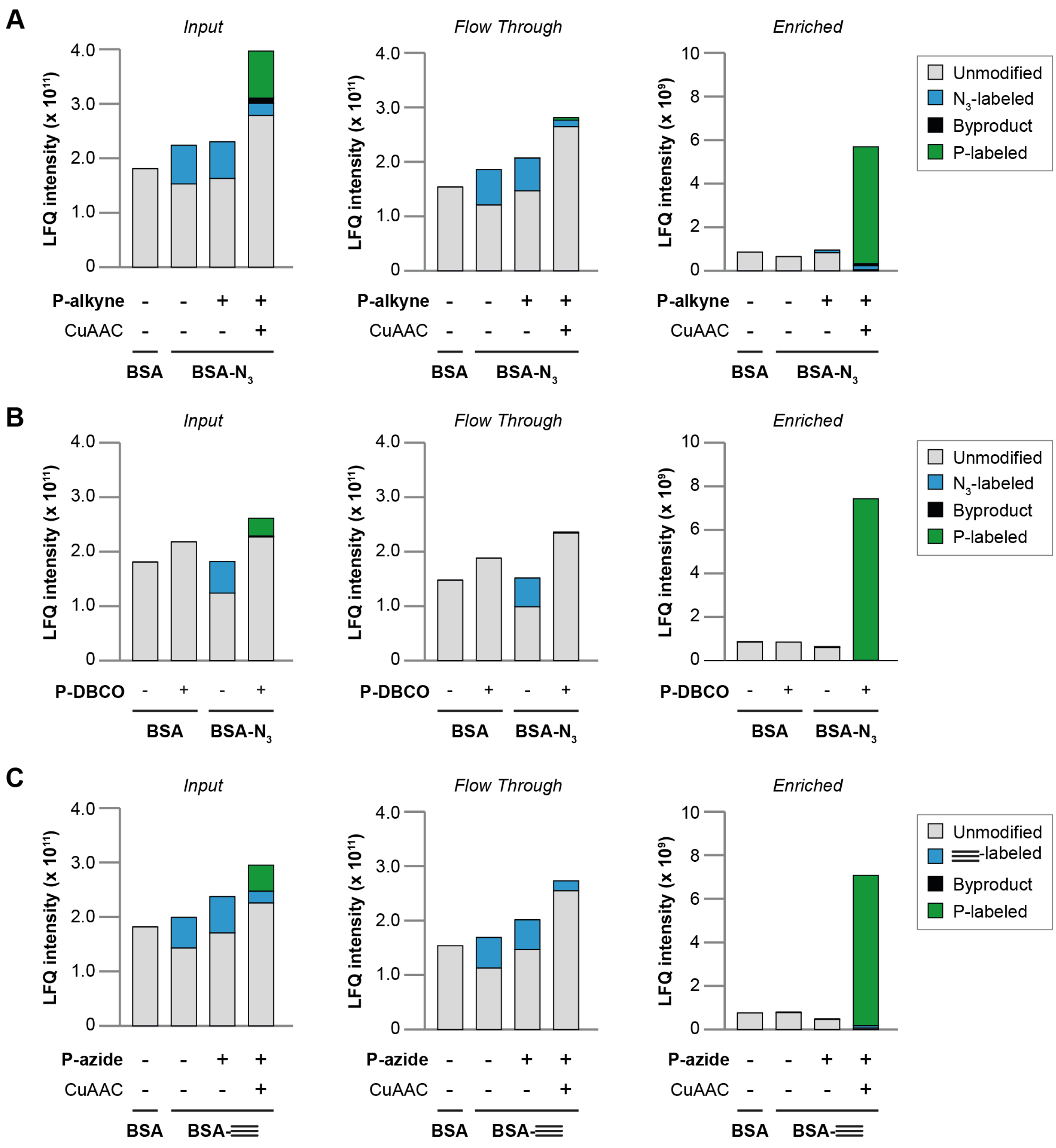

Supplementary Figure 2: PhosID is highly specific and efficient. BSA was functionalized at free amine groups of lysines with azide (BSA-N_3) or alkyne (BSA-≡) functionalities and clicked to the corresponding **(A) P-alkyne**, **(B) P-DBCO,** or **(C) P-azide** respectively. After trypsin digestion, phosphonate-modified peptides were retrieved by IMAC purification. Intensity of BSA peptides detected in IMAC input, flowthrough (FT) and elution (Enriched) fractions are plotted. Data derived from these panels are summarized in Figure 2A in the main manuscript. Source data provided in Source Data file.

Supplementary Table 1: Mixing ratios to functionalize BSA. For each experiment, the azide or alkyne NHS ester was used to incorporate the azide or alkyne functionality in 100 µg (about 1.5 nmol) of BSA, which contained 59 lysines. If not indicated, the ratio of 1:1 was used for modification of BSA.

Ratio lysine:NHS ester	Amount lysines in BSA (nmol)	Amount NHS ester added to reaction (nmol)	Stock of NHS ester used (mM)
1:5	88	440	20
1:1	88	88	4
1:0.2	88	17.6	0.8
1:0.04	88	3.5	0.16
1:0.02	88	1.76	0.08

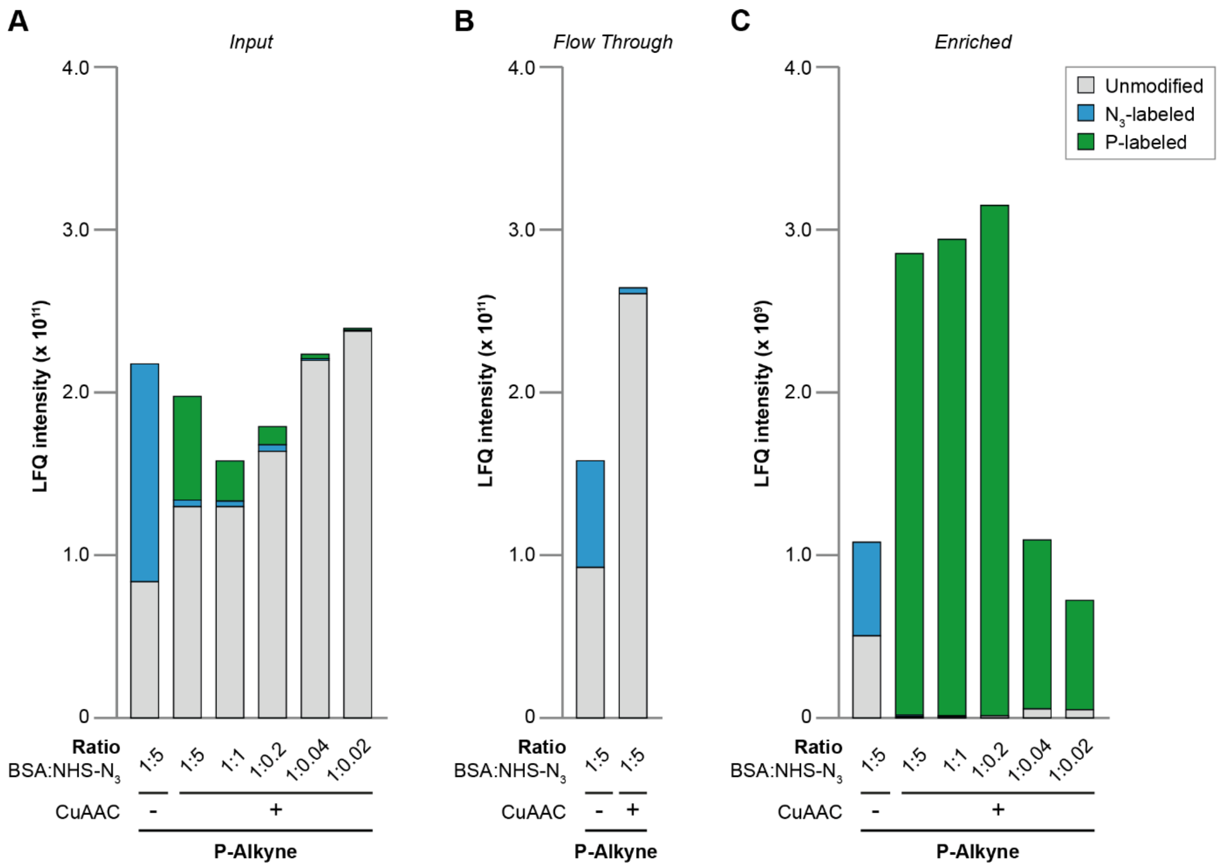

Supplementary Figure 3: The specificity of IMAC is retained in low azide to BSA ratio. BSA was labeled with decreasing amounts of azide moieties by changing the amount of Azide NHS ester (NHS-N_3). The ratio BSA:NHS-N_3 is described in Supplementary Table 1 and is based on the molar quantity of azide NHS ester and the number of lysine residues present in BSA. The CuAAC reaction was then performed with BSA-N_3 in the absence or presence of **P-alkyne** followed by digestion and IMAC enrichment. The IMAC input fraction **(A)** as well as the flow through **(B)** and elution **(C)** fractions were analyzed by LC-MS/MS with matched injection loads, and searched separately against the BSA fasta sequence. Source data provided in Source Data file.

Supplementary Table 2: Recovery of $BSA-N_3$ from a HeLa cell lysate by IMAC enrichment was highly sensitive. In tests of enrichment "Sensitivity", decreasing amounts of $BSA-N_3$ was spiked in 100 µg of a HeLa lysate, followed by the PhosID protocol using **P-alkyne**. In tests of "Recovery", 1 µg of $BSA-N_3$ was diluted in different amounts of HeLa lysate (10 or 1000 µg). Shown below are the intensities of unmodified peptides, phosphorylated peptides and PhosID peptides.

Ratio	$BSA-N_3$ (µg)	HeLa lysate (µg)	Unmodified peptides - Relative intensity (%)	Phosphorylated peptides - Relative intensity (%)	P-labeled peptides with P-alkyne - Relative intensity (%)	P-labeled peptides with P-alkyne - Counts
**Sensitivity**						
-	100	0	0.3	0	99.7	98
1:10	10	100	4.2	0.1	85.7	77
1:100	1	100	7.7	0.6	91.7	86
1:1,000	0.1	100	60.7	5.1	34.2	65
1:10,000	0.01	100	87.7	6.8	5.5	38
**Recovery**						
1:10	1	10	21.5	0.2	78.3	77
1:1,000	1	1,000	14.6	10.2	75.2	72

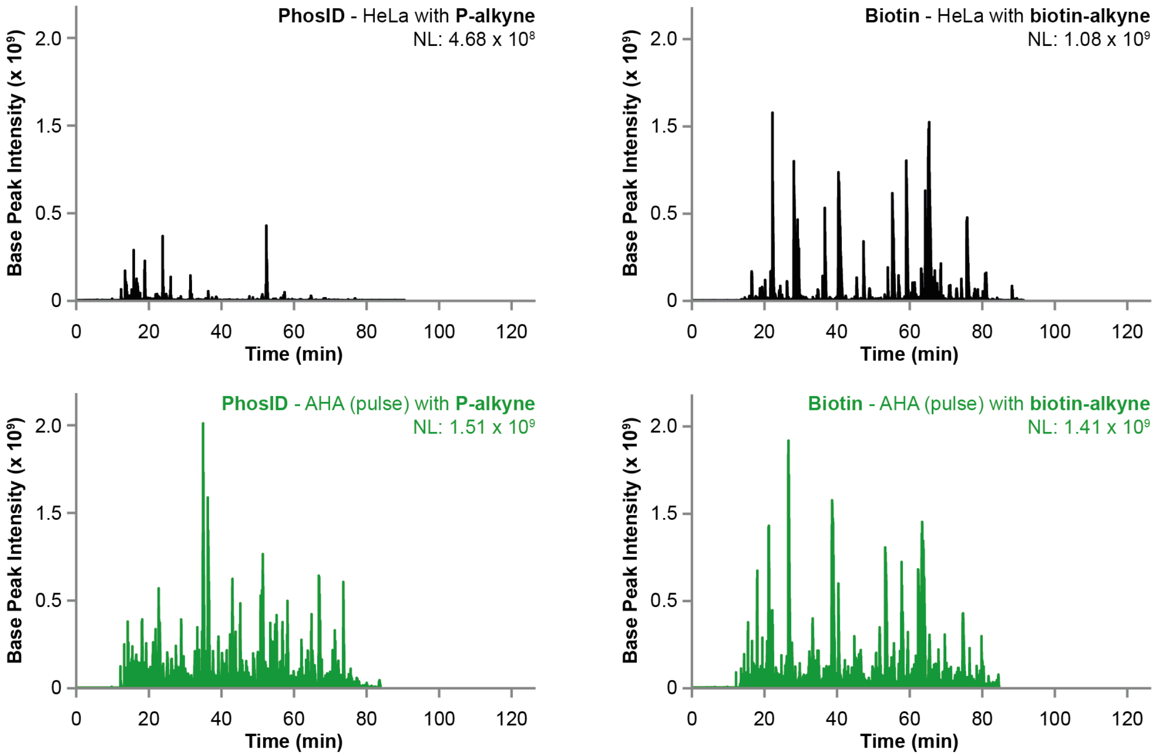

Supplementary Figure 4: LC-MS raw intensity trace from HeLa or AHA-pulse labeled HeLa cells after PhosID using P-alkyne or biotin enrichment using biotin-alkyne. All basepeak intensity traces are shown on the same intensity scale. PhosID enrichment from unlabeled HeLa digest gave a much lower background than the biotin-streptavidin approach (comparison of black traces), whereas the basepeak intensities from phosphonate-labeled HeLa digest were comparable (comparison of green traces).

Supplementary Table 3: Identification summary of PhosID and Biotin-streptavidin workflows. HeLa or HeLa labeled with AHA for 24 hours (AHA pulse) or about three weeks (AHA stable) were enriched using PhosID reacted with **P-alkyne** or Biotin workflow using **biotin-alkyne** (see Figure 3A for workflow). The data was searched against the human database, including the phosphonate-label or biotinylation as a variable modification. Data based on 3 experimental replicates. A peptide or protein was considered valid if identified in at least two out of three replicates. Parts of this table are featured in Figure 3C in the main text. Protein ID information is provided in Supplementary Table 4 in a separate excel document. Although the Biotin-streptavidin experiments retrieved around 17,000 peptides, already a background of 12,000 peptides was detected from HeLa lysates not labeled with Biotin-alkyne. This background was not a result of identified streptavidin peptides (Supplementary Table 6). Less than 3.5 % of the intensity included peptides from streptavidin in HeLa samples and less than 0.5 % of the intensity were streptavidin peptides in case of HeLa – AHA (pulse) and HeLa – AHA (stable).In the PhosID experiments on the other hand, the background of unmodified peptides was extremely low.

	**Modified**		**Unmodified**	
	**Peptides**	**Proteins**	**Peptides**	**Proteins**
**PhosID**				
HeLa	0	-	1,629	648
HeLa - AHA (pulse)	6,636	2,366	654	110
HeLa - AHA (stable)	6,155	2,259	579	104
**Biotin**				
HeLa	0	-	12,162	2,031
HeLa - AHA (pulse)	12	17	18,001	2,584
HeLa - AHA (stable)	10	16	16,966	2,541

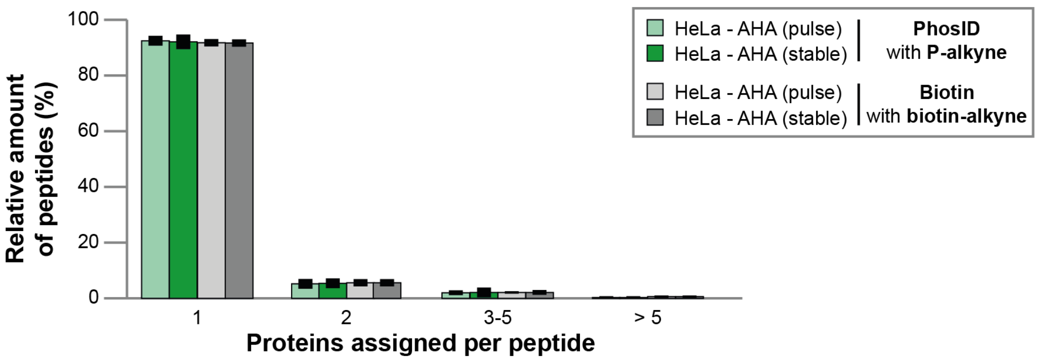

Supplementary Figure 5: The majority of the peptides were unambiguous and assigned to one protein for both PhosID using P-alkyne and biotin enrichment using biotin-alkyne. Histogram showing the relative number of proteins assigned per unique peptide for pulse and stable AHA labeled cells followed by PhosID or biotin enrichment. The total number of peptides identified in each experiment was scaled to 100%, standard deviation is based on three replicates. Error bars indicate standard deviation (*n*=3). Source data provided in Source Data file.

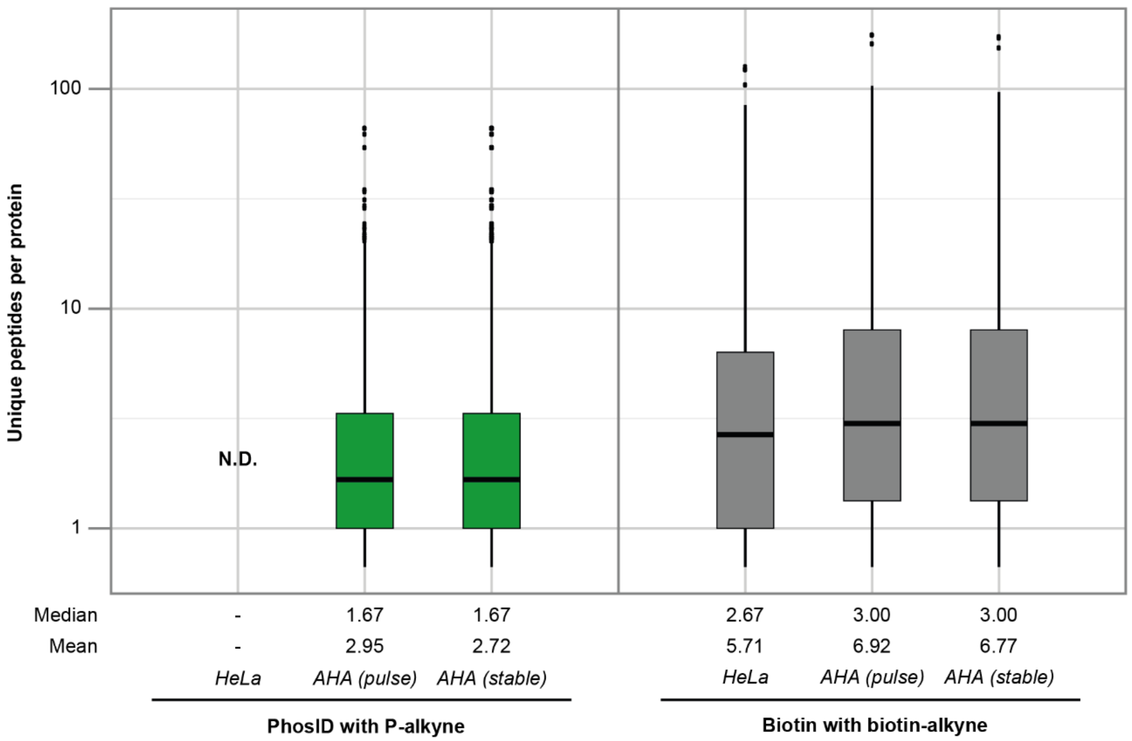

Supplementary Figure 6: The median and average number of unique peptides identified per protein is slightly lower in PhosID using P-alkyne than Biotin-streptavidin using biotin-alkyne. Boxplot for the unique peptides assigned to a protein, which was identified in at least two or more of the replicates of each experiment. Depicted are the median and mean for these samples. N.D., not detected. Source data provided in Source Data file.

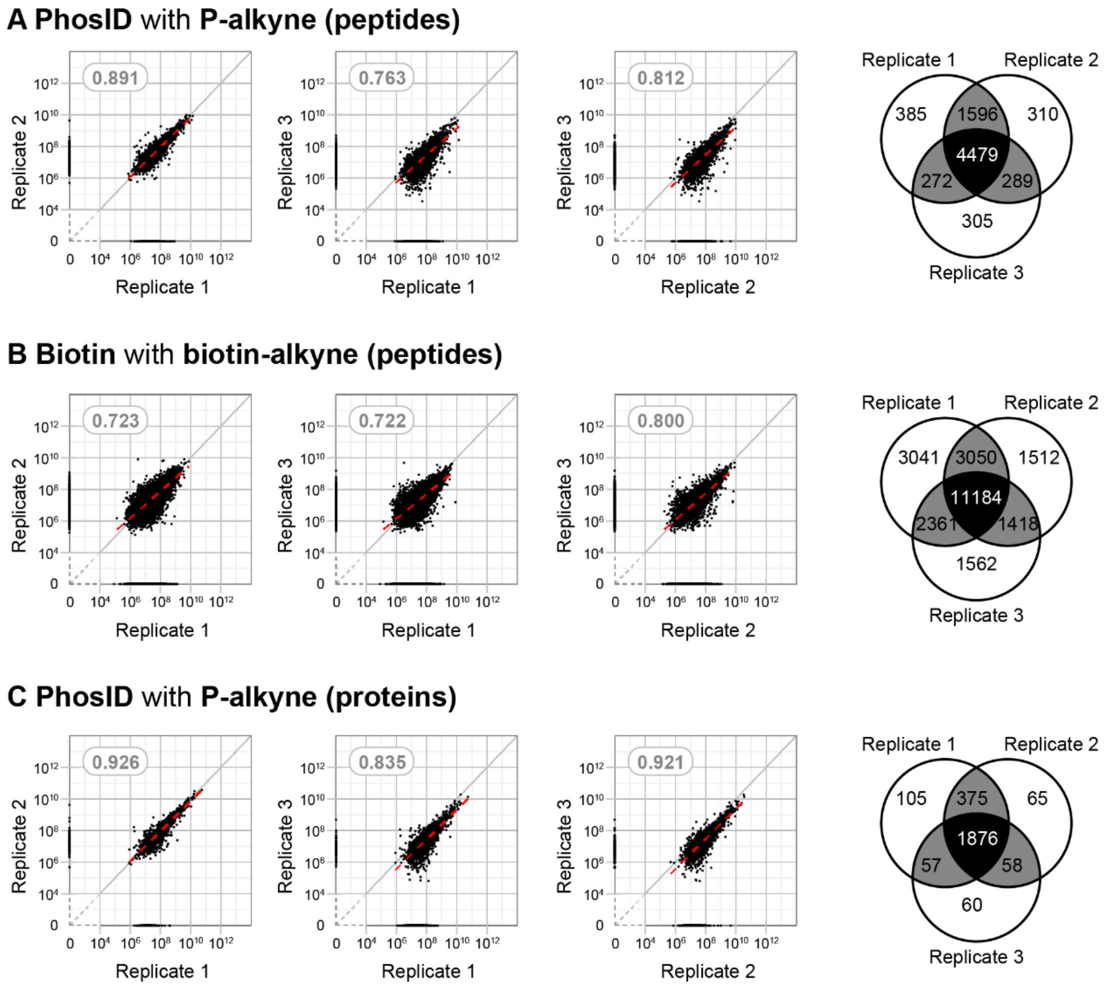

Supplementary Figure 7: Reproducibility within replicates of PhosID using P-alkyne or Biotin-streptavidin enrichment using biotin-alkyne. The intensity of the **(A)** phosphonate-labeled peptides, **(B)** biotin enriched peptides and **(C)** phosphonate-labeled proteins were plotted versus each other of the three AHA-pulse labeled replicate experiments. Pearson R^2 correlations were based on linear regression models and were exceeding 0.7 in all comparisons. In addition, the Venn diagrams on the right show the qualitative overlap in peptide or protein identifications.

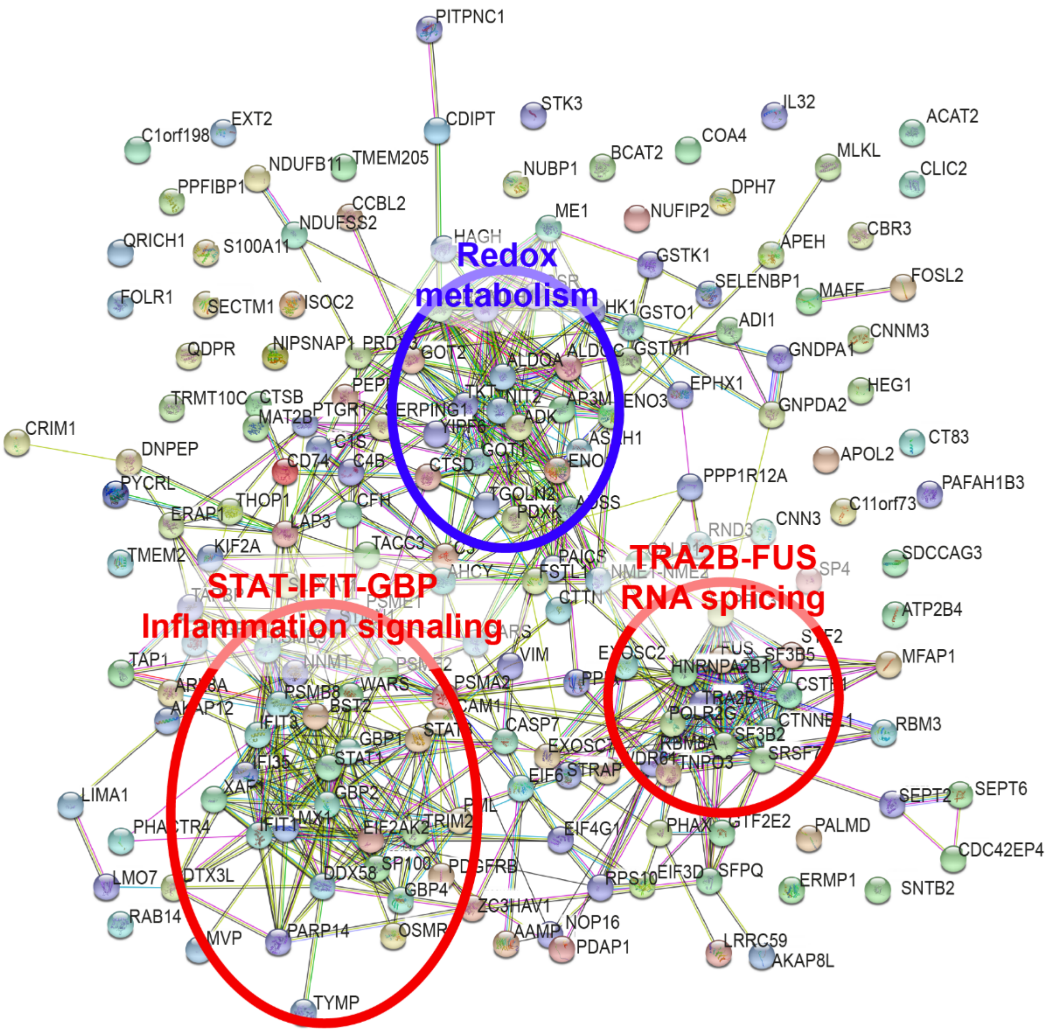

Supplementary Figure 8: STRING network of NSPs induced by 24h IFNγ treatment in HeLa cells. Redox and metabolic processes appear to be suppressed while proteins needed for RNA splicing and inflammation signaling are synthesized significantly more. Clusters indicated in red are induced, whereas clusters indicated in blue are suppressed by IFNγ stimulation.

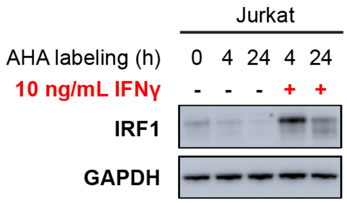

Supplementary Figure 9: Induction of IRF1 protein synthesis in Jurkat cells. Source data provided in Source Data file.
