# Supplementary material for: Fishing for newly synthesized proteins with phosphonate-handles
Source: Nat Commun. 2020 Jun 26;11:3244. doi: 10.1038/s41467-020-17010-0 (PMC7320153; doi:10.1038/s41467-020-17010-0)
Supplement: Supplementary file 4 — Description of Additional Supplementary Files [file 41467_2020_17010_MOESM4_ESM.pdf]

Description of Additional Supplementary Files

Name: Supplementary Data 1
Description: Protein List HeLa_Pulse_Stable

Name: Supplementary Data 2
Description: Streptavidin peptide identified

Name: Supplementary Data 3
Description: Protein List related to Main Figure 3

Name: Supplementary Data 4
Description: Protein List related to IFN treatment
